# BioPred: an R package for biomarkers analysis in precision medicine

**DOI:** 10.1093/bioinformatics/btae592

**Published:** 2024-10-07

**Authors:** Zihuan Liu, Yan Sun, Xin Huang

**Affiliations:** Data and Statistical Sciences, AbbVie Inc., North Chicago, IL, 60064, United States; Data and Statistical Sciences, AbbVie Inc., North Chicago, IL, 60064, United States; Data and Statistical Sciences, AbbVie Inc., North Chicago, IL, 60064, United States

## Abstract

**Summary:**

The R package BioPred offers a suite of tools for subgroup and biomarker analysis in precision medicine. Leveraging Extreme Gradient Boosting (XGBoost) along with propensity score weighting and A-learning methods, BioPred facilitates the optimization of individualized treatment rules to streamline subgroup identification. BioPred also enables the identification of predictive biomarkers and obtaining their importance rankings. Moreover, the package provides graphical plots tailored for biomarker analysis. This tool enables clinical researchers seeking to enhance their understanding of biomarkers and patient population in drug development.

**Availability and implementation:**

The package is available at CRAN and https://github.com/deeplearner0731/BioPred.

## 1 Introduction

Biomarkers are important tools for precision medicine in clinical trials development. Prognostic biomarkers provide an insights into patient disease outcomes, while predictive biomarkers predict treatment effects (Sechidis *et al.* 2018). Utilizing prognostic and predictive biomarkers, many subgroup identification methods are developed to explore patient populations heterogeneities in terms of disease progression and treatment response. For example, high PD-L1 IHC expression in patients with advanced non-small cell lung cancer is associated with improved efficacy of pembrolizumab (Garon *et al.* 2015), and BRCA1/2 mutations are instrumental in identifying patients likely to respond to PARP inhibitors (Ledermann *et al.* 2012). Numerous packages dedicated to subgroup analyses and predictive biomarker identification in clinical trials are available within the R statistical software environment. Notable examples include the MMMS (Li *et al.* 2014), GUIDE (Loh *et al.* 2015), ROWSi (Xu *et al.* 2015), subgroup (Schou and Marschner 2015), quint (Dusseldorp *et al.* 2016), SubgrpID (Huang *et al.* 2017), sparsereg (Ratkovic and Tingley 2017), TSDT (Battioui *et al.* 2018), FindIt (Egami *et al.* 2018), model4you (Seibold *et al.* 2019), SIDES (Riviere 2021), credsubs (Schnell *et al.* 2020), rlearner (Nie and Wager 2021), and CAPITAL (Cai *et al.* 2022).

In addition to the aforementioned packages, recent studies has demonstrated that a wide range of established statistical methodologies for subgroup identification fall under modified loss framework (Chen *et al.* 2017). Within this framework, the personalized R package have designed for optimal individualized treatment rule (ITR) estimation (Huling and Yu 2021). However, this package currently lacks integration with Extreme Gradient Boosting (XGBoost) within the modified loss function framework. Given XGBoost's widespread adoption in data mining communities, and its successful applications in biomedical domains, including prognostic biomarker identification (Li *et al.* 2022), there is a critical need for an R package specifically tailored to leverage XGBoost for the identification of subgroup and predictive biomarkers.

In this work, we introduce the R package BioPred, which integrates the modified loss function framework (Chen *et al.* 2017) with XGBoost to identify predictive biomarkers. In addition, BioPred provides a workflow for subgroup and predictive biomarker analysis. Specifically, the BioPred package offers analytical and visualization tools, including graphical representations of treatment effects in subgroups, biomarker response relationships, biomarker treatment interaction relationships in association with outcomes. Furthermore, BioPred is capable of handling commonly used endpoint types in clinical trials, including continuous, binary, and time-to-event endpoints. In addition, this package incorporates commonly used functions for biomarker analysis in clinical trials, such as cutoff analysis of biomarkers for responder enrichment. Overall, this platform allows researchers to conduct exploratory biomarker analyses effectively for the pharmaceutical industry.

## 2 BioPred R package

**Figure 1.**
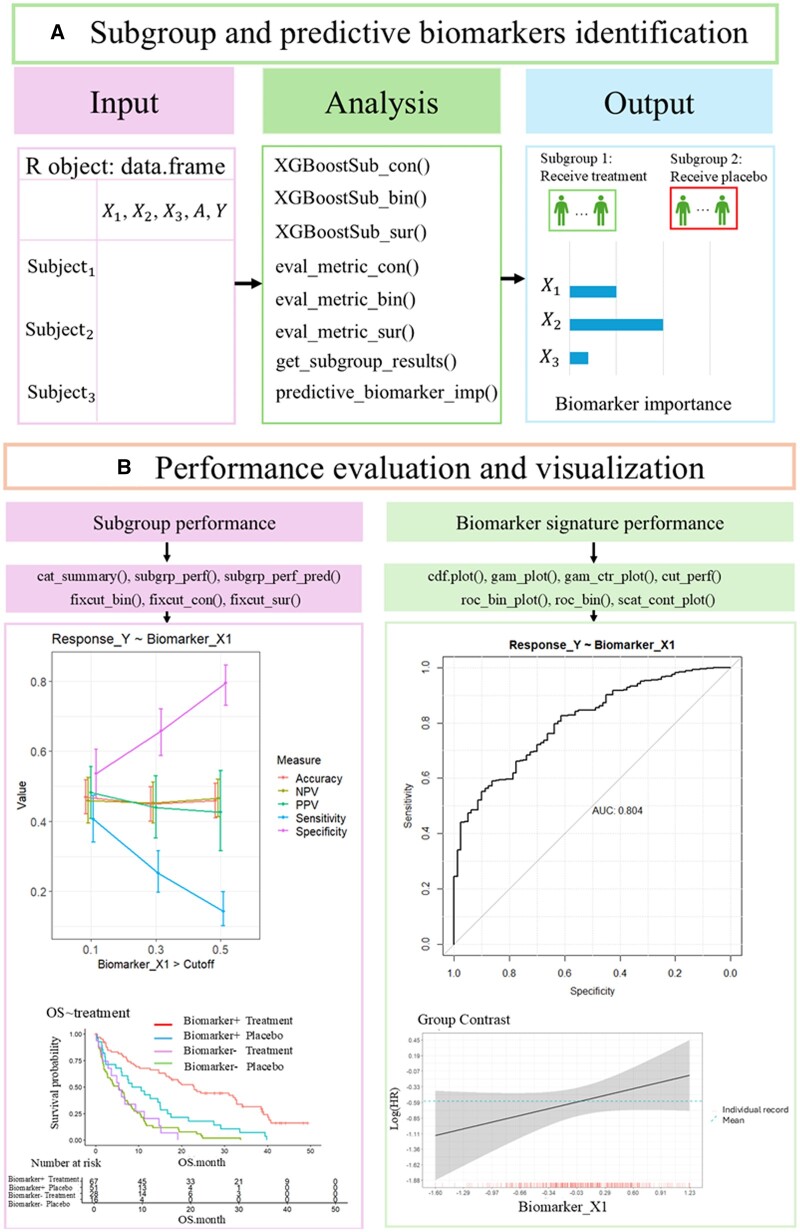
Analysis and visualization tools using the BioPred R package. (A) Subgroup and predictive biomarker identification: this panel illustrates the use of several key functions-XGBoostSub_con(), XGBoostSub_bin(), XGBoostSub_sur(), eval_metric_con(), eval_metric_bin(), eval_metric_sur(), get_subgroup_results(), and predictive_biomarker_imp()-to identify subgroups and predictive biomarkers. The input is an R DataFrame, with the output including predicted subgroup results and a ranked list of biomarker importance. (B) Performance evaluation and visualization: this component demonstrates performance evaluation and visualization, utilizing functions such as cat_summary(), subgrp_perf(), subgrp_perf_pred(), fixcut_bin(), fixcut_con(), fixcut_sur(), cdf.plot(), gam_plot(), gam_ctr_plot(), cut_perf(), roc_bin_plot(), roc_bin(), and scat_cont_plot(). The input for this component comprises results from (A), with these functions providing insights into the strength of the identified biomarkers' association with outcomes. “Biomarker +” denotes the biomarker-positive subpopulation, where the biomarker value meets the cutoff or other criteria, while “Biomarker –” signifies the biomarker-negative subpopulation, where the biomarker value does not meet the respective criteria.

The general workflow of the package is shown in Fig. 1. BioPred combines A-learning (Murphy 2003) and Weight-learning techniques (Chen *et al.* 2017) with XGBoost to optimize ITR for both subgroup and predictive biomarker identification. The package accommodates different endpoint types, including continuous, binary, and time-to-event endpoints, making it versatile in clinical application. Results generated by BioPred can be presented in table or through visually graphical plots, facilitated by dedicated functions within the package. Furthermore, BioPred is poised to integrate simulation functions, enabling the generation of simulation data to augment analytical capabilities. We present a list of all BioPred functions along with their respective descriptions in Supplementary Table S1.

### 2.1 The model

We denote the observed data by $\{(X_{i},\;{\;A}_{i}{,\;Y}_{i}$), i=1,…,N} consisting of n independent patients, where $Y_{i}$ denotes outcome, $X_{i}\;\mathrm{and}\;A_{i}\;$represents covariate of biomarkers and treatment assignment for i*th* subjects. We adopt the Neyman–Rubin potential outcome framework in causal inference (Rubin 1978, Rubin 2005). In this framework, only one of the potential outcomes can be observed, i.e. $Y_{i}$ =12(1+Ai) Yi(1) +12 (${1-A}_{i})\;Y_{i}(-1)$, where $Y_{i}\left(1\right)\;$and $Y_{i}\left(-1\right)$ are the potential outcomes if the patient i receives a treatment $\left(A_{i}=1\right)$ and a placebo $\left(A_{i}=-1\right)$, respectively. Let (X,A,Y) denote identically distributed copies of observed data, the completely unspecified regression model is formulated as follows:

E(Y│A,X)=Z(X)A+H(X)

where Z(X)=1/2 [E(Y│A=1,X)-E(Y│A=-1,X)] is a contrast function that reflects treatment effects given X. A recent study proposes a generalized loss function framework for estimating the monotone transformation of $Z\left(X\right)$, denoted by f(X) (Chen *et al.* 2017). This framework includes propensity score A-learning and weight-learning methods, as provided by:

LA=1N ∑i=1NM{Yi,(Ai+1)/2- πXi×f(Xi)}

and

LW=1N∑i=1NM{Yi,Ai×fXi}AiπXi+(1-Ai)/2

where π(X)=P(A=1│X) is the propensity score, and $M\left(y,v\right)$ is required to meet the following conditions: (i) $M\left(y,v\right)$ is convex in v and (ii) $V\left(y\right)≔M\left(y,0\right)$ is monotone in y. These requirements are sufficient for Fisher consistent subgroup identification (Qian and Murphy 2011, Zhao *et al.* 2012). Table 1 lists the options for M(y,v) function. We employ XGBoost to construct $\hat{f}$ based on $L\left(f\right)$

**Table 1.** List of M(y,v) function.

Outcome (Y)	Descriptions
Continuous	$(v-y{)}^{2}$
Binary	$-[yv-\log\left(1+e^{-v}\right)]$
Time-to-event	$-\{\int_{0}^{\tau }(v-\log\left[E\left\{e^{v}I\left(Z\geq u\right)\right\}])dN(u)\right\}$ ^a^

a $Y=\left(Z,\delta \right)=\left\{\tilde{Z}\wedge C,I\left(\tilde{Z}\leq z\right)\right\},$ where $\tilde{Z}$ is the survival time, C is the censoring time, $N(t)=I(\tilde{Z}\leq t)\delta$, and τ is a fixed point such that $P\left(Z\geq \tau \right)>0$.

### 2.2 Subgroup identification

The *XGBoostSub_con*(), *XGBoostSub_bin*(), and *XGBoostSub_sur*() functions facilitate the development of subgroup models across various outcome types, with flexibility in selecting the appropriate M function. These models are constructed using the *xgb.train()* function from the xgboost package (Chen and Guestrin 2016). Please see the documentation for the *xgb.train()* function of the xgboost package for more details on the possible arguments. The *censors* argument to denote the censor status of the data. The *trt* argument represents the integer vector of observed treatment statuses (e.g. for binary treatment, 1 or −1 in the ith position indicates patient i received the treatment or placebo). Additionally, the loss argument corresponds to the type of M function, with options including *loss*=A-learning and *loss*=weight-learning. The *propensity.score* argument refers to a vector of values ranging between 0 and 1, representing the propensity scores. The output of *get_subgroup_results()* include subgroup results based on the optimal ITR, indicating whether patients will receive treatment or placebo. The value of the modified loss can be obtained using *eval_metric_bin()*, *eval_metric_con()*, and *eval_metric_sur()*.

### 2.3 Predictive biomarker selection

The function *predictive_biomarker_imp()* can be used to access importance rankings for identified predictive biomarkers. Specifically, this function operates by taking the output of XGBoost-based subgroup functions as input and returns the importance ranking of the biomarkers along with importance plots. Of note, biomarker importance is evaluated using the *xgb.importance()* function within the xgboost package (Chen and Guestrin 2016).

### 2.4 Performance evaluation and visualization for subgroup and biomarker analysis

BioPred offers essential visualization and post-hoc analysis functions integral to biomarker analysis in clinical practice. The functions *cdf.plot()* empower users to visually explore the cumulative distribution of an individual biomarker in different subgroups. Typically employed after biomarker identification, the function aids in quantifying the percentage of subjects falling below or above specified cutoff values. Furthermore, the function *roc_bin_plot()* allows users to generate Receiver Operating Characteristic (ROC) plots for binary outcomes associated with individual biomarkers. Additionally, the functions *fixcut_bin(), fixcut_con()*, and *fixcut_sur()* facilitate the evaluation of various performance metrics across a range of candidate cutoffs for different outcome types. These functions assist users in selecting the cutoff of a biomarker based on metrics such as Youden index, Kappa agreement, *P*-value of subgroup testing specified by the user. This capability is particularly valuable for companion diagnostics (CDx) development with limited candidate cutoffs (e.g. IHC). Moreover, functions such as *subgrp_perf_pred()*, *subgrp_perf* (), and *cut_perf ()* serve to evaluate subgroup performance. These functions are commonly utilized in both predictive and prognostic biomarker analyses, providing valuable insights into the strength of the identified biomarker's association with outcomes. This also aids clinical scientists in determining the optimal cutoff points for informed clinical decision-making.

### 2.5 Illustrative example

Following the BioPred workflow (Fig. 1), we demonstrate the application of binary outcomes using a tutorial dataset containing 10 biomarkers $x_{i},i=1,\ldots ,10.$ To develop the XGBoostSub_bin model with the A-learning loss, we employ the following approach:

>model<-*XGBoostSub_bin(X, y, trt, pi,Loss_type = “A_learning”, params = list(learning_rate = 0.01, max_depth = 1, lambda = 5, tree_method = ‘hist’), nrounds = 300, disable_default_eval_metric = 0, verbose = FALSE)*

Next, the subgroup results can be obtained using:

>pred<-*get_subgroup_results (model, X)*

The A-learning loss metrics can be obtained as follows:

>loss<-*eval_metric_bin (model, X, y, pi, trt, Loss_type = “A_learning”)*

The importance ranking for identified predictive biomarkers can be accessed through:

>prd_imp<-*predictive_biomarker_imp(model)*

If the output indicates that $x_{1}$is the most important biomarker, the optimal cutoff for this biomarker can be determined by:

>*fixcut_bin (yvar=“y”, xvar=“x1”, dir=“>“, cutoffs=c(0.1,0.3,0.5), data=tutorial_data, method=“Fisher”, yvar.display=“y”, xvar.display=“Biomarker x1”, vert.x=F)*

Here, the list c(0.1,0.3,0.5) represents candidate optimal cutoff values. Readers can adjust this list based on their dataset. For instance, if an optimal cutoff of 0.5 is determined, the performance at this cutoff value can be assessed using the following code:

>*res=cut_perf (yvar=“y”, censorvar=NULL, xvar=“x1”, cutoff=c(0.5), dir=“>”, xvars.adj=NULL, data=tutorial_data, type=“c”, yvar.display=“y”, xvar.display=“Biomarker x1”).*

Once the optimal cutoff is determined, the “biomarker-positive” group can be defined. This allows for the classification into four groups: placebo biomarker-positive, placebo biomarker-negative, treatment biomarker-positive, and treatment biomarker-negative. The predictive model performance based on these defined subgroups can then be assessed using:

>*res = subgrp_perf_pred(yvar=“y.time”, censorvar=“y.event”, grpvar=“biogroup”, grpname=c(“biomarker_positive”,‘biomarker_negative’),trtvar=“treatment_categorical”, trtname=c(“Placebo”, “Treatment”), xvars.adj=NULL,data=tutorial_data, type=“s”)*

In addition, the following method can be employed to evaluate whether a selected biomarker is predictive:

>*gam_ctr_plot (yvar=“y.time”, censorvar=“y.event”, xvar= “x1”, xvars.adj=NULL,sxvars.adj=NULL,trtvar=“trt”,type=“s”,data=tutorial_data, k=5, title=“Group Contrast”, ybreaks=NULL, xbreaks=NULL, rugcol.var=NULL,link.scale=T, prt.sum=T, prt.chk=F, outlier.rm=F).*

Finally, to determine whether the identified biomarker is prognostic, the association between the binary response variable y and the biomarker $x_{1}$ can be examined using:

>*roc_bin_plot (yvar=“y”, xvars=“x1”, dirs=“auto”, data=tutorial_data, yvar.display=“y.bin”, xvars.display=“Biomarker x1”)*

## 3 Conclusion

Traditionally, the effect of predictive biomarkers is estimated by fitting a regression model that includes an interaction between the biomarker and treatment. However, such parametric models with interaction terms often struggle to identify predictive biomarkers due to the “curse of dimensionality.” By incorporating XGBoost with modified loss function, our BioPred R package overcomes these limitations, enabling the optimization of ITRs for subgroups, identifying predictive biomarkers, selecting optimal biomarker cutoffs, and visualizing results graphically, even as the number of biomarkers increases with data from genomics and proteomics. Furthermore, BioPred holds practical clinical applications, including the development of CDx and enrichment designs for clinical trials.

## Supplementary Material

btae592_Supplementary_Data

## Data Availability

No new data were generated or analysed in support of this research.

## References

[btae592-B1] Battioui C , Denton B, Shen L. TSDT: Treatment-specific subgroup detection tool. In: *R Package Version*. 2018.

[btae592-B2] Cai H , Lu W, Marceau West R et al CAPITAL: optimal subgroup identification via constrained policy tree search. Stat Med 2022;41:4227–44. 35799329 10.1002/sim.9507 PMC9544117

[btae592-B3] Chen S , Tian L, Cai T et al A general statistical framework for subgroup identification and comparative treatment scoring. Biometrics 2017;73:1199–209. 28211943 10.1111/biom.12676 PMC5561419

[btae592-B4] Chen T , Guestrin C. Xgboost: A scalable tree boosting system. In: *Proceedings of the 22nd ACM SIGKDD International Conference on Knowledge Discovery and Data Mining*, San Francisco, California, USA, August 13-17, 2016. New York, NY, United States: Association for Computing Machinery, 2016, 785–94.

[btae592-B5] Dusseldorp E , Doove L, Mechelen I. Quint: an R package for the identification of subgroups of clients who differ in which treatment alternative is best for them. Behav Res Methods 2016;48:650–63. 26092391 10.3758/s13428-015-0594-z PMC4891398

[btae592-B6] Egami N , Ratkovic M, Imai K. *FindIt: Finding Heterogeneous Treatment Effects.* R package available at CRAN. 2018.

[btae592-B7] Garon EB , Rizvi NA, Hui R et al; KEYNOTE-001 Investigators. Pembrolizumab for the treatment of non–small-cell lung cancer. N Engl J Med 2015;372:2018–28. 25891174 10.1056/NEJMoa1501824

[btae592-B8] Huang X , Sun Y, Trow P et al Patient subgroup identification for clinical drug development. Stat Med 2017;36:1414–28. 28147447 10.1002/sim.7236 PMC7704099

[btae592-B9] Huling JD , Yu M. Subgroup identification using the personalized package. J Stat Softw 2021;98:1–60.

[btae592-B10] Ledermann J , Harter P, Gourley C et al Olaparib maintenance therapy in platinum-sensitive relapsed ovarian cancer. N Engl J Med 2012;366:1382–92. 22452356 10.1056/NEJMoa1105535

[btae592-B11] Li K , Yao S, Zhang Z et al Efficient gradient boosting for prognostic biomarker discovery. Bioinformatics 2022;38:1631–8. 34978570 10.1093/bioinformatics/btab869 PMC10060728

[btae592-B12] Li L , Guennel T, Marshall S et al A multi-marker molecular signature approach for treatment-specific subgroup identification with survival outcomes. Pharmacogenomics J 2014;14:439–45. 24637498 10.1038/tpj.2014.9

[btae592-B13] Loh WY , He X, Man M. A regression tree approach to identifying subgroups with differential treatment effects. Stat Med 2015;34:1818–33. 25656439 10.1002/sim.6454 PMC4393794

[btae592-B14] Murphy SA. Optimal dynamic treatment regimes. J R Stat Soc Ser B Stat Methodol 2003;65:331–55.

[btae592-B15] Nie X , Wager S. Quasi-oracle estimation of heterogeneous treatment effects. Biometrika 2021;108:299–319.

[btae592-B16] Qian M , Murphy SA. Performance guarantees for individualized treatment rules. Ann Stat 2011;39:1180–210. 21666835 10.1214/10-AOS864 PMC3110016

[btae592-B17] Ratkovic M , Tingley D. Sparse estimation and uncertainty with application to subgroup analysis. Polit Anal 2017;25:1–40.

[btae592-B18] Riviere M-K. Sides: subgroup identification based on differential effect search. 2021. https://cran.r-project.org/web/packages/SIDES/SIDES. pdf

[btae592-B19] Rubin DB. Bayesian inference for causal effects: the role of randomization. Ann Stat 1978;6:34–58.

[btae592-B20] Rubin DB. Causal inference using potential outcomes: design, modeling, decisions. J Am Stat Assoc 2005;100:322–31.

[btae592-B21] Schnell PM , Fiecas M, Carlin BP. Credsubs: multiplicity-adjusted subset identification. J Stat Soft 2020;94:1–22.

[btae592-B22] Schou IM , Marschner IC. Methods for exploring treatment effect heterogeneity in subgroup analysis: an application to global clinical trials. Pharm Stat 2015;14:44–55. 25376518 10.1002/pst.1656

[btae592-B23] Sechidis K , Papangelou K, Metcalfe PD et al Distinguishing prognostic and predictive biomarkers: an information theoretic approach. Bioinformatics 2018;34:3365–76. 29726967 10.1093/bioinformatics/bty357 PMC6157098

[btae592-B24] Seibold H , Zeileis A, Hothorn T. model4you: an R package for personalised treatment effect estimation. JORS 2019;7:17.

[btae592-B25] Xu Y , Yu M, Zhao Y-Q et al Regularized outcome weighted subgroup identification for differential treatment effects. Biometrics 2015;71:645–53. 25962845 10.1111/biom.12322 PMC5395466

[btae592-B26] Zhao Y , Zeng D, Rush AJ et al Estimating individualized treatment rules using outcome weighted learning. J Am Stat Assoc 2012;107:1106–18. 23630406 10.1080/01621459.2012.695674 PMC3636816

