# Supplementary material for: BioPred: an R package for biomarkers analysis in precision medicine
Source: Bioinformatics. 2024 Oct 7;40(10):btae592. doi: 10.1093/bioinformatics/btae592 (PMC11483108; doi:10.1093/bioinformatics/btae592)
Supplement: btae592_Supplementary_Data [file btae592_supplementary_data.docx]

**Supplementary materials for BioPred: an R package for biomarkers analysis in precision medicine**

Zihuan Liu, Yan Sun and Xin Huang

Data and Statistical Sciences, AbbVie Inc., North Chicago, IL;

**Table 1.** List of functions implemented in BioPred package

| Function | Description |
| --- | --- |
| XGBoostSub_con | XGBoost model with modified loss function for subgroup identification with continuous outcomes |
| XGBoostSub_bin | XGBoost model with modified loss function for subgroup identification with binary outcomes |
| XGBoostSub_sur | XGBoost model with modified loss function for subgroup identification with survival outcomes |
| eval_metric_con | Evaluation metrics for XGBoostSub-con model |
| eval_metric_bin | Evaluation metrics for XGBoostSub-bin model |
| eval_metric_sur | Evaluation metrics for XGBoostSub-sur model |
| predictive_biomarker_imp | Summarizes predictive biomarker importance based on XGBoost-based subgroup models |
| get_subgroup_results | Predicts the treatment assignment for each patient |
| cdf_plot | Cumulative distribution plot (CDF) plot for individual biomarker |
| roc_bin_plot | Receiver operating characteristic (ROC) curve for different biomarkers associated with binary outcome |
| roc_bin | Generates a table containing the AUC values for different biomarkers associated with binary outcome |
| scat_cont_plot | Generates a scatter plot for exploring the relationship between a continuous response variable and a biomarker variable |
| gam_plot | Generates a generalized additive model (GAM) plot for exploring the relationship between response and biomarker |
| gam_ctr_plot | Computes and plots the contrasts between treatment and control group based on a GAM for exploring the relationship between treatment benefit and biomarker |
| fixcut_con | Fixed cutoff analysis for individual biomarker associated with continuous outcome |
| fixcut_bin | Fixed cutoff analysis for individual biomarker associated with binary outcome |
| fixcut_sur | Fixed cutoff analysis for individual biomarker associated with survival outcome |
| cut_perf | Evaluates the performance of predictive model at a selected cutoff point |
| cat_summary | Summarize categorical biomarkers for subgroups defined by a list of categorical variables |
| subgrp_perf_pred | Subgroup performance evaluation for a predictive biomarker |
| subgrp_perf | Subgroup performance evaluation for a prognostic biomarker |
